# Sensitisation of HeLa Cell Cultures to Xanthone Treatment by RNAi-Mediated Silencing of NANOG and STAT3

**DOI:** 10.3390/cimb47070529

**Published:** 2025-07-09

**Authors:** Oliwia Gruszka, Dorota Żelaszczyk, Henryk Marona, Ilona Anna Bednarek

**Affiliations:** 1 Department of Biotechnology and Genetic Engineering, Faculty of Pharmaceutical Sciences in Sosnowiec, Medical University of Silesia, 40-055 Katowice, Poland; oliwia.gruszka@sum.edu.pl; 2 Department of Bioorganic Chemistry, Chair of Organic Chemistry, Faculty of Pharmacy, Jagiellonian University Medical College, 30-688 Krakow, Poland; dorota.zelaszczyk@uj.edu.pl (D.Ż.); henryk.marona@uj.edu.pl (H.M.)

**Keywords:** xanthones, α-mangostin, gambogic acid, STAT3, NANOG, RNAi, cancer

## Abstract

The increasing morbidity of various types of cancer in the world’s population and the limited number of universal methods of their treatment contribute to the growth in research into the development of new treatment strategies. Most of this research focuses on treatments that target specific factors in cancer cell signalling pathways. There is also great interest in drugs derived from natural substances, as these represent one of the largest sources of potential pharmaceuticals. In our analysis, we focused on the action of α-mangostin and gambogic acid, which are natural xanthones or their synthetic derivatives. We studied their influence on the expression of STAT3 and NANOG, which play a confirmed role in different stages of cancer development. For this purpose, we applied RNAi-mediated gene silencing of NANOG and STAT3 to enhance the efficacy of xanthone-based anticancer treatment in HeLa cell cultures. After stimulating the cells with xanthones, we determined the expression of the tested transcription factors and the ROS level. In addition, we determined the cytotoxicity and apoptosis of the cells. Our research results confirm the anticancer efficacy of the analysed xanthones and demonstrate the role of the tested transcription factors. Silencing these factors makes cancer cells more susceptible to xanthone treatment.

## 1. Introduction

Cancer is a significant health concern that leads to a high number of deaths around the world. One of the hallmarks of cancer cells is their heterogeneity, which results from genetic and epigenetic changes [1].

Even when appropriate treatment strategies are adopted for specific types of cancer, new solutions or alternative pathways are constantly sought to improve treatment effectiveness and/or reduce the adverse effects of anticancer therapies. Natural compounds are a constant source of new drugs, including those synthesised based on the known structure and activity of natural compounds. One group of compounds of importance in cancer therapy is xanthone compounds.

Xanthones are oxygenated heterocyclic compounds whose chemical structure is based on a dibenzo-γ-pyrone skeleton [2]. It is reported that xanthones display a range of activities, including anticancer [3], antibacterial, antifungal [4], antiviral, antidiabetic, anti-inflammatory [2], anti-allergic, and neuroprotective activities [3]. The anticancer activity of their derivatives may be dependent on the type, number, and location of the functional groups [2]. Over the past dozen years, hundreds of xanthones have been isolated from natural sources, and many of their derivatives have been synthesised [5].

Natural xanthones have been isolated from higher plants belonging to more than 20 families, as well as from fungi, bacteria, and lichens. *Garcinia mangostana* (mangosteen) is one of the richest sources of natural xanthones [2]. One of them is α-mangostin (MAG), which has been found to display a number of biological activities. For instance, MAG is known to have strong antiproliferative, anti-metastatic, and pro-apoptotic properties in cancer cells, and it has also been observed to induce cytotoxic effects [3]. Another anticancer xanthone, gambogic acid (GA), has been isolated from a tropical tree, Garcinia hanburyi [6]. It should be noted that both MAG and GA appear to exert their activity through a number of different mechanisms, including the stimulation of caspases, reactive oxygen species (ROS), the disruption of mitochondrial membrane potential, and the release of cytochrome c [3,6]. MAG and GA have also been shown to have a role in reducing cancer invasion and angiogenesis [7,8]. Their activity has been confirmed in many types of cancer, e.g., blood, skin, colon [8], lung, breast, pancreas [3,6,9], prostate, and bone [6]. It is important to emphasise that many cancers are caused by abnormalities in signalling pathway transduction, resulting in the promotion of tumour cell proliferation and expansion. As a rule, modulation of signal transduction in cancer cells activates selected transcription factors, which in turn promote the expression of genes that drive tumourigenesis and metastasis.

The discovery of the transcriptional factor NANOG and the establishment of its role in the self-renewal and pluripotency of stem cells were significant milestones in contemporary science. It has been suggested by several studies that NANOG may have a role to play in the development of tumours. This has been shown to include an influence on proliferation, migration, and invasion, as well as on the emergence of chemoresistance and immune tolerance [10,11]. It has been observed that NANOG expression increases in many tumours derived from various tissues, including breast, cervix, ovary, prostate, head and neck, kidney, lung, central nervous system, and gastrointestinal tract [12,13,14,15]. It has also been suggested that it plays a key role in leukaemia, as well as colorectal and pancreatic cancers [10]. It has been observed that there appears to be a significantly higher expression of NANOG in cancer stem cells (CSCs) than in other tumour cells [13]. This observation is associated with the maintenance of CSC characteristics through signalling pathways such as TGF-β, Wnt/β-catenin, JAK/STAT, Notch, and Hedgehog, which show a positive correlation with the expression of NANOG [16]. Secondary tumour foci consist of large quantities of actively proliferating CSCs, which may help to maintain high levels of NANOG [17]. It has been observed that stem cells and transcription factors such as OCT-4, SOX-2, or NANOG play a role in the development of chemoresistance in cancer cells, particularly CSCs [18]. A very important fact is that NANOG is also an element of the molecular pathways contributing to the development of chemoresistance, e.g., NANOG/TCL1/Akt [19] and NANOG/STAT3/MDR1. The formation of NANOG-STAT3 heterocomplexes activates the expression of the MDR1 gene encoding P-gp, the principal cell membrane transporter responsible for xenobiotic efflux [11,20,21]. The silencing of NANOG expression by RNA interference (RNAi) leads to cell cycle arrest, inhibition of tumour growth, enhancement of apoptosis, inhibition of cancer cell migration and invasion [22,23], and an increase in chemotherapy effects [23].

Signal transducers and activators of transcription (STATs) are part of a group of transcription factors that play a role in signal transduction, a process that responds to hormones, cytokines, growth factors, and other polypeptide ligands. The STAT family consists of seven cytoplasmic proteins activated by phosphorylation [24]. It has been reported that STATs play an important role in many physiological processes. However, excessive signalling based on these factors, mainly STAT3 and STAT5, has been noted in different cancers [25]. It has been reported that STAT3 plays a key role in the development of epithelial tumours and immune proliferative disorders [26]. STAT3 can be activated not only by cytokines, such as IL-6 or IFN, and by the JAK signalling pathway or oncogenic proteins [27] but also by numerous environmental agents like UV radiation, tobacco smoke, or infections [28]. The phosphorylation of STAT3 initiates the transcription of many genes, including genes responsible for oncogenesis and tumour progression [29]. In particular, mitochondrial activation through the phosphorylation of serine (Ser727) is thought to promote tumour growth and metastasis. It also participates in inflammation and interactions between tumour and normal cells, and it is thought to be an important mediator of tumour immune evasion [27]. Therefore, inhibition of STAT3 not only inhibits the growth of cancer cells but also may increase the anticancer immune response [24,29].

As described above, both transcription factors play a significant role in cancer cells. Furthermore, there appears to be cross-talk between NANOG and STAT3 signalling [16,17]. It is known that NANOG and STAT3 can form heterocomplexes, which bind to and synergistically activate STAT3-dependent gene promoters [21]. Additionally, there is some evidence to suggest a connection between NANOG expression and the process of enforced phosphorylation of STAT3 [10,14,21]. It is possible that STAT3 has the capacity to regulate NANOG expression through an epigenetic mechanism [20].

An interesting and important phenomenon associated with cervical cancer is the link between tumour formation and papillomavirus (HPV) infection. Furthermore, it has been observed that the activation of STAT3 factor is correlated with HPV infection in a group of cervical cancers [30]. Advances in prevention are important, not least the search for new therapeutic solutions incorporating low-cost, safe strategies based on or derived from natural compounds. Based on our previous observations of the potential use of natural xanthones such as MAG and GA, and taking into account their limitations such as poor solubility, a short half-life and instability, we focused our research on the use of synthetic xanthone derivatives to verify their anticancer potential to compare their selected activities to natural compounds.

Taking the above into account, our work aimed to evaluate the effect of natural xanthones (MAG and GA) and synthetic xanthone derivatives on selected anticancer properties in HeLa cells, paying particular attention to their effect on cells with native and RNA interference-silenced STAT3 and NANOG gene expression.

## 2. Materials and Methods

### 2.1. Chemicals

Agarose, DAPI (4′,6-diamidino-2-phenylindole dihydrochloride), DMSO (dimethyl sulfoxide), gambogic acid (GA), H_2_DCFDA (2′,7′-dichlorodihydrofluorescein diacetate), α-mangostin (MAG), N-acetyl-L-cysteine (NAC), and trypan blue were purchased from Sigma-Aldrich, St. Louis, MO, USA. Bradford Reagent was purchased from ThermoFisher Scientific, Waltham, MA, USA. Media and chemicals used for in vitro cell cultivation were purchased from ThermoFisher Scientific.

### 2.2. Synthesis and Purification of Synthetic Xanthone Derivatives

**Figure 1:**
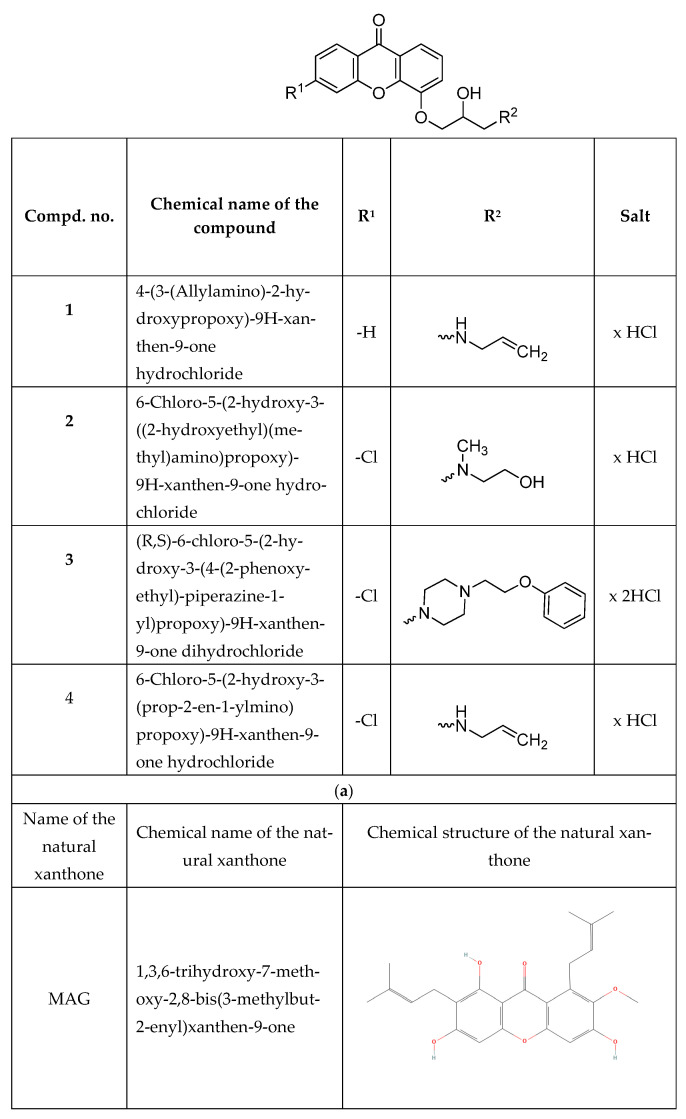

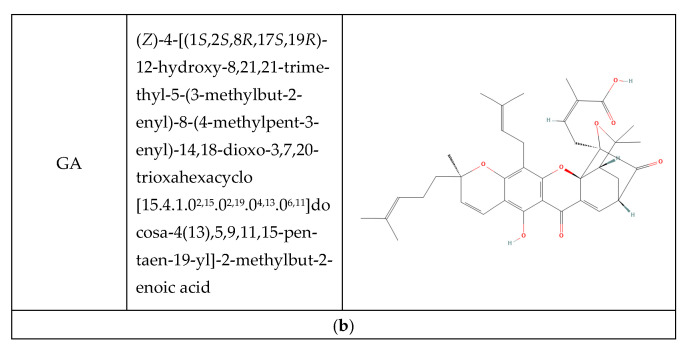
Chemical structure of (**a**) synthetic derivatives and (**b**) natural xanthone [32] evaluated in this study.

A detailed methodology for the synthesis, purification, and physicochemical properties of xanthone derivatives has been described in our previous paper [31]. The synthesized compounds were subjected to ^1^H and ^13^C NMR analyses as well as mass spectrometry. The melting points of all compounds were also determined. The purity was confirmed by thin-layer chromatography. All compounds used in the study are summarised in Figure 1.

### 2.3. Cell Culture and Treatment Conditions

All analyses were carried out on HeLa (ATCC^®^ CCL-2™) cells. Cell cultures were routinely propagated at 37 °C/5% CO_2_ in RPMI-1640 supplemented with 10% FBS (Foetal bovine serum) and gentamicin. Xanthones were dissolved in water or in DMSO and stored at −20 °C until analysis. All treatments were performed for 12 h under non-cytotoxic conditions, unless stated otherwise. IC50 values were established in our previous paper [31]. Each compound was investigated in three independent experiments; each experiment was carried out in triplicate. For cell proliferation, cytotoxicity, and apoptosis determination, all compounds were used at IC25 concentrations.

### 2.4. Detection of Reactive Oxygen Species (ROS)

Intracellular ROS were measured based on the detection of fluorescent product DCF (2′,7′-dichlorofluorescein) yielded by the oxidation of the H_2_DCFDA probe. After the treatments, the growth medium was removed, cells were washed with D-PBS (Dulbecco’s Phosphate Buffered Saline), and then 500 µL of medium containing 5 µM H_2_DCFDA was added to each well. Cells were incubated for 1 h. Then, the medium was removed, and cells were washed with D-PBS, scraped, and subjected to flow cytometry analysis (BD FACSAria II).

### 2.5. Plasmids and Transfections

The pSUPER RNAi System has been used to cause the gene-specific down-regulation of expression (OligoEngine). This system provides a mammalian expression vector that directs intracellular synthesis of siRNA-like transcripts. The resulting transcript of the recombinant vector is predicted to fold back on itself to form a 19-base pair stem-loop structure (shRNA); the stem-loop precursor transcript is quickly cleaved in the cell to produce a functional siRNA (small interfering RNA). Details on the design and preparation of the recombinant plasmids have been published previously [33,34]. The siRNA and its corresponding shRNA sequences were designed using *sirna* computer software (version 6.6.0 EMBOSS program). Vectors encoding interference RNA molecules targeting gene transcripts STAT3 and NANOG were designed and synthesised. Control sequences in transfecting cells were also used: nonsense sequence SCR (scrambled), which was not complementary to any of the human genes. Large-scale plasmid preparations were obtained by alkaline lysis and purification on chromatographic columns (Endo-free Plasmid Maxi kit, Qiagen, Venlo, The Netherlands). For transfections, cells were seeded in 24-well plates (5 × 10^4^ cells per well) in RPMI-1640 containing 10% FBS, without antibiotics. The following day, cell cultures were transfected with suitable plasmids using Lipofectamine 2000 (ThermoFisher Scientific). Control transfections with pEF/myc/cyto/GFP (ThermoFisher Scientific) were carried out using the same conditions. GFP (Green fluorescent protein) was visualized under a fluorescence inverted microscope, Axiovert 40 CFL (Zeiss, Oberkochen, Germany). RNAi efficiency was estimated using a control plasmid expressing an shRNA targeting GFP mRNA.

### 2.6. RNA Extraction

Total RNA was extracted using TRI-Reagent (Sigma-Aldrich). RNA extracts were treated with DNase I and purified using Direct-zol RNA MiniPrep (Zymo Research, Irvine, CA, USA). RNA concentration was determined spectrophotometrically (λ = 260 nm). RNA integrity was estimated by using 2% agarose gel electrophoresis with Gel-Red™ staining (Merck, Rahway, NJ, USA).

### 2.7. Real-Time™ RT-PCR

Transcript levels of NANOG and STAT3 were determined by SYBR Green Real-Time™ RT-PCR assays. The expression of the studied genes was normalized to the endogenous control (GAPDH mRNA) by the 2^−∆∆Ct^ method. One-step Real-Time™ RT-PCR assays were carried out using an Mx3000P thermal cycler (Stratagene, La Jolla, CA, USA). All reagents were purchased from Stratagene. The thermal profile was 50 °C for 30 min (reverse transcription), then 95 °C for 10 min, 40 two-step cycles of 94 °C for 15 s and 55 °C for 30 s, and 72 °C for 10 min (Real-Time™ PCR), followed by a dissociation protocol (60–95 °C; 30 min). The specificity of PCR products was confirmed by analysis of their dissociation curves after each Real-Time™ RT-PCR amplification.

### 2.8. ELISA

The expression of NANOG and STAT3 was confirmed by ELISA assays: Human homeobox protein NANOG ELISA Kit (MyBioSource, San Diego, CA, USA) and STAT3 Total PhosphoTracer ELISA Kit (Abcam, Cambridge, UK). Cell lysates for STAT3 detection were obtained by lysis of 4 × 10^3^ cells in a commercial buffer. Fluorometric detection was carried out using a 548/555 nm filter. For NANOG detection, cell lysates were obtained from 10^6^ cells suspended in 100 μL of PBS and homogenized by repeated freezing–thaw (2 cycles) followed by sonication (Sonics Vibra-Cell, Newtown, CT, USA). Absorbance was measured at λ = 450 nm. NANOG standards were used to obtain a standard curve for absolute quantification.

### 2.9. Cell Proliferation

Cell proliferation was estimated based on EdU (5-ethynyl-2′-deoxyuridine) incorporation using the Click-iT EdU Alexa Fluor 488 Imaging Kit (ThermoFisher Scientific). Cells were seeded in 48-well culture plates at a density of 20 × 10^3^ cells and treated as indicated. EdU-positive cells were visualized using an inverted fluorescence microscope. Cell nuclei were counterstained with DAPI (4′,6-diamidino-2-phenylindole). In each culture well, 10 fields of view were photographed (at least 100 cells in each well).

### 2.10. Cytotoxicity and Apoptosis Determination

LDH release was determined in cell culture supernatants using CytoTox 96 Non-radioactive Cytotoxicity Assay (Promega). Absorbance was measured at λ = 490 nm. Results were calculated as Abs_sample_/Abs_LDH_ max × 100%. Apoptosis was evaluated by DNA fragmentation assay using the Cell Death Detection ELISA kit (Sigma-Aldrich). Absorbance was measured at λ = 405 nm. The results were calculated as EF (enrichment factor), based on the following formula: Abs_sample_/Abs_negative_ control.

### 2.11. Statistical Analysis

Quantitative data were compared by Student’s *t* test or Mann–Whitney U test. For multiple comparisons, ANOVA or ANOVA Kruskal–Wallis was used; *p* < 0.05 was considered statistically significant. All calculations were performed with Statistica v. 10.0 software.

## 3. Results

### 3.1. Expression of NANOG and STAT3 Under Xanthone Treatment

**Figure 2:**
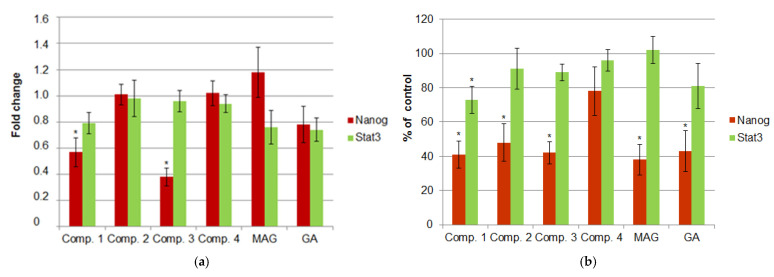
The expression of NANOG and STAT3 in HeLa cell cultures under xanthone treatment. (**a**) The relative mRNA levels of the studied genes calculated by the 2^−∆∆Ct^ method and expressed as median fold change (+/− interquartile range) compared to the calibrator (untreated). (**b**) The relative protein expression of NANOG and STAT3. The bars present the mean percentage (+/− stand. dev.) of the control (untreated). * indicates statistically significant differences vs. untreated (*p* < 0.05).

In the first part of this study, we explored the possibility of xanthone treatment influencing the expression of NANOG and STAT3. Expression was measured at both the mRNA and protein levels. The results can be found in Figure 2a,b. While no significant changes were observed in STAT3 expression under xanthone treatment (except for comp. 1, which decreased the level of STAT3 protein), NANOG expression was significantly reduced by most xanthones, including the natural (GA, MAG) and synthetic derivatives (except for comp. 4). Decrease in NANOG expression at the transcriptional level was statistically significant only in cell cultures treated with comp. 1 and 3. The cultures treated with MAG showed the highest decrease in the NANOG protein level (by 64.53%, *p* = 0.003). However, it is also worth noting that most of the tested xanthones inhibited NANOG expression with similar strength, and no significant differences between the compounds were observed. The absolute concentration of NANOG protein in untreated cell cultures was 19.37 pg/mL.

### 3.2. Induction of ROS in Xanthone-Treated Cells

**Figure 3:**
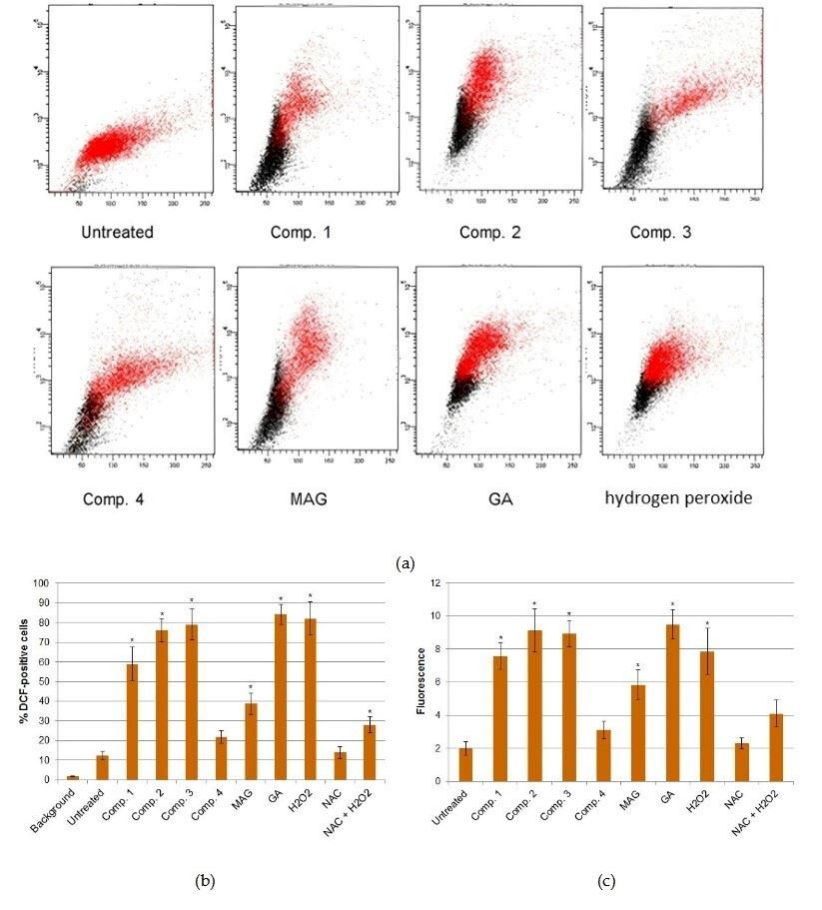
ROS determination in the xanthone-treated HeLa cell cultures. (**a**) Representative flow cytometry histograms. (**b**,**c**) Quantitative results of flow cytometry analyses. Bars present the mean value (+/− stand. dev.) of the percentage of DCF-positive cell (**b**) or the fluorescence values (**c**). * indicates statistically significant differences vs. the untreated control (*p* < 0.05).

In the interest of exploring potential reasons why the xanthone derivatives used in this study appeared to impact NANOG expression, we conducted an analysis of ROS levels in the examined cell cultures. ROS were determined by DCF detection using flow cytometry. Figure 3, which compares the average percentage of DCF-positive cells, as well as the mean fluorescence values, appears to indicate that most xanthones used in our study significantly increased ROS levels in HeLa cells. Additionally, these compounds induced ROS with a strength that was similar to that of a potent ROS-inducer, i.e., hydrogen peroxide. Only comp. 4 did not display significant pro-oxidant potential. Importantly, the xanthones that displayed ROS-stimulating potential were the same compounds that inhibited NANOG expression.

### 3.3. Knockdown of NANOG and STAT3 by shRNA-Mediated Gene Silencing

**Figure 4:**
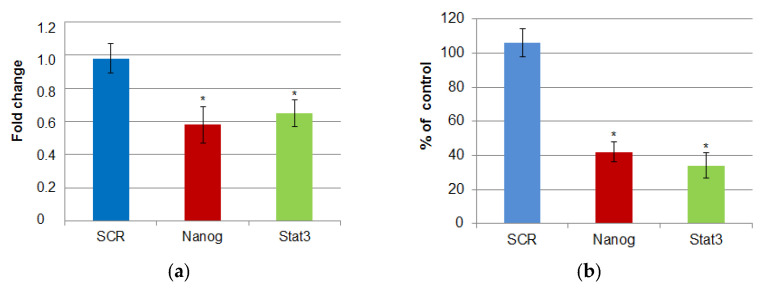
The expression of NANOG and STAT3 in HeLa cell cultures subjected to shRNA-mediated gene silencing. (**a**) The relative mRNA levels of the studied genes calculated by the 2^−∆∆Ct^ method and expressed as median fold change (+/− interquartile range) compared to the calibrator (untransfected). (**b**) The relative protein expression of NANOG and STAT3. The bars present the mean percentage (+/− stand. dev.) relative to the control (untransfected). * indicates statistically significant differences (*p* < 0.05).

The overall transfection efficiency was 94.01%, as estimated by the expression of the reporter protein (GFP). In an effort to evaluate the efficiency of shRNA-mediated gene silencing, cells were co-transfected with a pEF/myc/cyto/GFP plasmid and a pSUPER plasmid expressing anti-GFP shRNA. Control cultures were co-transfected with pEF/myc/cyto/GFP and a pSUPER plasmid expressing scrambled (non-coding) shRNA. The overall efficiency of GFP silencing was 87%. For NANOG and STAT3 silencing, gene-specific pSUPER plasmids were used. Expression levels of NANOG and STAT3 were then examined 48 h after the transfections. Gene-specific shRNA appeared to successfully block the expression of both genes at the mRNA and protein levels. The average silencing efficiencies were 42% for NANOG and 35% for STAT3 at the mRNA level (Figure 4a), while at the protein level, the efficiencies were 58% and 66%, respectively (Figure 4b).

### 3.4. Cell Viability, Proliferation, and Apoptosis After RNAi-Mediated Gene Silencing and Xanthone Treatments

To carry out the combination of RNAi and xanthone treatment, cell cultures were first transfected with the pSUPER plasmids expressing shRNA targeting NANOG or STAT3 transcripts. In addition, in all analyses, control transfections were carried out using plasmids expressing a non-targeting control (scrambled sequence) shRNA. The following day, treatments with the studied xanthone derivatives were performed under conditions described in the Materials and Methods Section.

**Figure 5:**
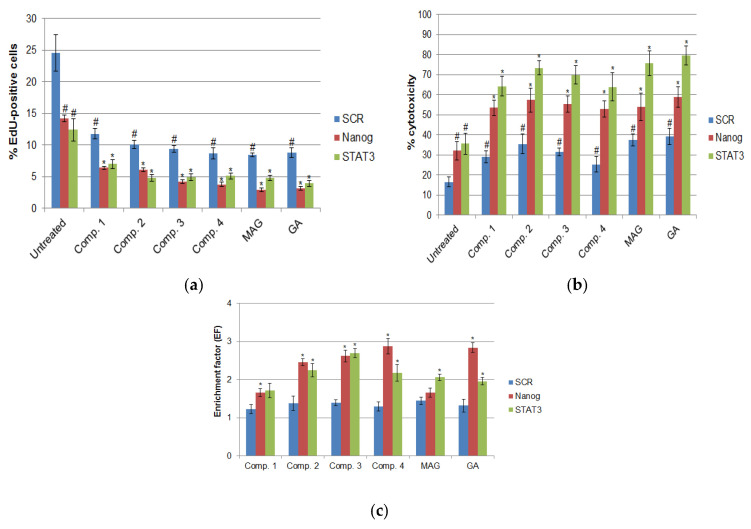
Proliferation, cytotoxicity, and apoptosis under xanthone treatment and gene silencing. In all graphs, * and # indicate statistically significant differences (*p* < 0.05); * vs. xanthone-treated, SCR-transfected; # vs. xanthone-untreated, SCR-transfected. (**a**) Proliferation measured as EdU-positive cell percentages (bars represent mean +/− stand. dev.). (**b**) Results of cytotoxicity evaluation measured as percentages of maximal LDH release (bars represent mean +/− stand. dev.). (**c**) Apoptosis evaluation expressed as EF (enrichment factor) compared to untreated controls (bars represent mean +/− stand. dev.).

Statistical analysis of EdU incorporation assays suggested that in all cultures, significant decreases in cell proliferation occurred with the combined use of xanthones and RNAi targeting either NANOG or STAT3 compared to cultures in which xanthone treatments were performed after control transfections (shSCR) (Figure 5a). It is notable that a significant decrease was also observed in cultures that were transfected with NANOG- or STAT3-targeting constructs, but not further treated with xanthones, or in cultures transfected with shSCR-expressing plasmids and then treated with xanthones. Inference suggests that both xanthone treatment and gene-specific RNA interference significantly inhibited cell proliferation, while the combination of the two agents resulted in increased efficacy in proliferation arrest. Furthermore, it was observed that both molecular targets, NANOG and STAT3, appeared to be equally effective in blocking HeLa cell proliferation.

The results were further supported by analysis of cytotoxicity and apoptosis. The level of cytotoxicity was determined by an LDH release assay. It was observed that in a variety of cultures, there was a notable increase in toxicity after RNAi-mediated silencing of NANOG or STAT3, as well as after the combined use of transfection and xanthone treatment (Figure 5b). It is interesting to note that, in most cases, STAT3 targeting by shRNA tended to result in higher levels of toxicity compared to NANOG silencing. The apoptosis rate was estimated using a DNA fragmentation ELISA. It seems that the combined use of xanthone derivatives and shRNA-mediated gene silencing led to an increase in DNA fragmentation compared to both control cultures (transfected with shSCR-expressing constructs) and untreated cultures, which were only subjected to NANOG or STAT3 silencing. Additionally, it appears that gene silencing alone may have resulted in a significant increase in the apoptosis rate (Figure 5c).

## 4. Discussion

Tumour diseases have the potential to pose a serious threat to public health. The most important challenges in medical sciences are perhaps the comprehension of the mechanisms leading to cancer development and the search for new, effective anticancer drugs. It has been suggested that a potential avenue for identifying novel therapeutic agents could be through the screening of natural compounds that have been utilised in traditional medicine. It is thought that plants belonging to the Clusiaceae family are rich in compounds termed xanthones, e.g., GA and MAG. The extensive utilisation of xanthones in traditional medicine, particularly in countries such as China, India, and Thailand, has led scientists to hypothesise that these compounds may possess potential in the study of the influence of xanthones on cancer cells [2,3,8,35]. Both gambogic acid (GA) and α-mangostin (MAG) block critical signalling pathways in cancer cells, activate apoptosis through signalling pathways [36,37], and exhibit anti-metastatic activity against various types of cancer. However, the clinical application of these natural xanthones is limited due to their poor bioavailability. Synthetic derivatives could improve the usefulness of these compounds.

In this study, we explored the potential of RNAi-mediated gene silencing of NANOG and STAT3 to enhance the efficacy of xanthone-based anticancer treatment of HeLa cell cultures. Additionally, we observed that xanthones themselves may influence the expression of NANOG.

Current investigations suggest that STAT3 and NANOG may have important roles at multiple stages of tumour progression. It has been demonstrated that both of these factors are often overexpressed in a variety of cancers [6,9,10,13,14], which may contribute to tumour growth, metastasis, chemoresistance [38], and immune evasion [26,27,28]. Additionally, it has been suggested that there is a certain functional redundancy and cooperation between STAT3 and NANOG [20]. To date, there is a lack of research papers indicating the direct inhibition of NANOG or STAT3 expression by xanthone derivatives. We demonstrated that almost all synthetic xanthone derivatives analysed in our study exerted a significant influence on NANOG expression, while no significant influence was demonstrated on STAT3 expression. In addition, it was observed that xanthones, which decreased NANOG expression, also demonstrated a notable oxidative potential, which resulted in an elevation of ROS levels. It has been suggested by Han et al. that ROS may influence NANOG expression in a p53-dependent manner. The generation of ROS leads to the nuclear translocation of p53, which could in turn inhibit NANOG expression and activate the mitochondrial apoptotic pathway [39]. Furthermore, the results of our study suggest that ROS stimulation by oxidative xanthones may be a mechanism of NANOG down-regulation. Similar observations have been made for rooperol, the main phenolic constituent of *Hypoxis hemerocallidea*. ROS induction by rooperol contributes to the deregulation of p53, leading to the inhibition of OCT-4, NANOG, and SOX-2 expression in CSCs [40]. Although there is currently no direct evidence regarding the influence of xanthones on STAT3 expression, the existing literature suggests that some xanthone derivatives may modulate STAT3 phosphorylation or interfere with the cellular pathways regulated by activated STAT3. It also appears that Xu et al. proved that two natural GA derivatives significantly decrease STAT3 signalling in cancer cells through JAK2 inhibition, which subsequently leads to a decrease in STAT3 phosphorylation [35]. Prasad et al. indicated that GA inhibits phosphorylation of STAT3 in U266 myeloma cells, which leads to the impairment of STAT3 dimerization and subsequent nuclear translocation [41]. Shan et al. confirmed that the antitumor properties of MAG could be partially attributed to blockade of STAT3 activity [42].

Since its discovery in 1998, the technology of RNA interference has been applied in research and therapy, as shown by the increasing number of clinical trials each year, including studies on RNAi use in cancer therapy [43]. We explored the potential of RNAi as a therapeutic strategy in combination with xanthone treatment, aiming to contribute to our understanding of its effectiveness. It has been observed that STAT3 down-regulation has the potential to enhance the anticancer activity of various chemotherapeutic agents, either through gene-specific molecular techniques [44,45,46] or by pharmacological modulation [46,47]. It is known that STAT3-directed siRNA has been used to sensitise breast cancer cells to doxorubicin [42] and neuroblastoma cells to cisplatin [48]. Hong et al. demonstrated the clinical efficacy of antisense oligonucleotide targeting STAT3 in lung cancer and lymphoma therapies [49]. Other investigations have suggested that small-molecule inhibitors might be useful in targeting STAT3, for example, cucurbitacin B, which has been used to make laryngeal carcinoma cells more sensitive to cisplatin [50]. It is also worth flagging that RNA interference has been applied in NANOG silencing to reduce the proliferation [22,51], migration, and metastasis of cancer cells [33]. RNAi-mediated NANOG knockdown has been successfully used to improve the chemosensitivity of HepG2 liver cancer cells to doxorubicin [52] or oesophageal cancer cells to cisplatin [53]. It also appears that an indirect approach was used, which involved the down-regulation of ROR (HOX Transcript Antisense Intergenic RNA), which led to the inhibition of NANOG expression. This, in turn, resulted in a decrease in proliferation and invasion of pancreatic cancer cells [54]. RNAi has been used to indirectly diminish P-gp-mediated chemoresistance through NANOG knockdown [20]. Zhou et al. explored a method of sensitising cancer cells to cisplatin, which involved the suppression of STAT3/MDR1 molecular pathways through silencing the long non-coding RNA HOTAIR [55].

In our study, we used xanthone derivatives modified with amines. As we have previously touched upon, the anticancer activity of xanthones appears to be related to the type, number, and position of functional groups attached to the xanthone skeleton [2]. It has been suggested by a previous study [31] that the type and arrangement of the side chain have an influence on the anticancer activity of the compound. Derivatives with substituents in the C2 position of the xanthone core may not be as effective as those with substituents in the C4 position when it comes to their impact on HeLa cell lines. For this reason, they were selected for this study. In an attempt to draw some comparisons between the activity of the tested compounds and natural xanthones, gambogic acid was used in this study as a natural equivalent to synthetic compounds **1**–**4**. The choice of gambogic acid was based on the presence of a functional group (4-oxa-tricyclo[4.3.1.0^3,7^]dec-2-one ring) in the C4 position. The reference sample was α-mangostin, which does not have any functional group at this position. It appears that compounds **1**–**4** contain an allyl moiety in the C4 position (compounds **1** and **4**), an N-methylethanolamine moiety (compound **2**), or a functional group containing a piperazine ring in its structure (compound **3**). Piperazine is found in many natural compounds and in many drugs. Piperazine provides beneficial effects on bioavailability and solubility, which is why it can improve the biological effect of the compound [56]. The analysis suggests that there is no clear association between the presence of a functional group in the C4 position of the xanthone core and its effect on the reduction in NANOG and STAT3 expression. This study also analysed the effect of molecules differing only in the chlorine atom substituent at the C6 position (compounds **2**–**4**). It is our understanding, based on a comparison of the activity of compounds **1** and **4**, that the substituent in the C6 position has the potential to significantly reduce their inhibitory effect on the expression of both STAT3 and NANOG.

Based on our results, we can suggest that the depletion of NANOG or STAT3 through RNAi results in the sensitization of cancer cells to therapeutic agents. In our study, we have examined the possibility of enhancing the efficiency of xanthone-based anticancer treatment through RNAi-mediated silencing of NANOG and STAT3. Gene-specific RNAi-pretreated cell cultures appeared to be more sensitive to xanthone treatment than the control cultures. The aberrant expression, in addition to the activation, of both inducible and ubiquitous transcription factors has been observed to be a frequent occurrence in the process of carcinogenesis. Information is available on the effect of STAT3 expression on the presence and characteristics of stem cells in cervical cancer [57]. Moreover, in the case of cervical carcinogenesis, a strong correlation was found between HPV infection and STAT3 activation. In the case of cervical cancer cells, where the integration of HPV genes into the cell genome is additionally observed, STAT3 activation occurs, with tumour-promoting effects [30]. The simultaneous “knockdown” of such cancer cells by silencing the expression of STAT3, which interacts independently with the transcription factor NANOG, and treatment of the cells with xanthone-derived compounds leads to the activation of mechanisms, including ROS-dependent mechanisms, leading to the elimination of the cancer cells. It seems that a combination of RNAi and xanthone treatments may lead to a significant inhibition of proliferation and an increase in apoptosis. Our results support the idea of the usefulness of the chemosensitisation of cancer cells using molecular targeting, especially in reference to the connection between STAT3/NANOG activation in the cervical cancer HeLa cell model.

### Strengths and Limitations

The overexpression of STAT3, or the re-expression of NANOG, has been observed in a variety of cancer cells. Researchers aim to block the molecular pathways that facilitate cancer cell survival and proliferation by developing inhibitors or modulators of STAT and NANOG activity.

A substantial body of literature indicates a strong association between the malignant phenotype of cervical cancer cells, including cancer stem cells, and the altered expression of STAT3 and NANOG. Despite the presence of divergent data regarding the sporadic ineffectiveness of the suppression of STAT3 activity, it is imperative to acknowledge that strategies that disrupt STAT3 signalling can nevertheless elicit substantial antitumour effects by modulating the tumour microenvironment.

The approach adopted in this study is a two-pronged strategy that involves the silencing of both STAT3 and NANOG, which are transcription factors that have been shown to cooperate in the maintenance of cancer cell progression and chemoresistance. The present study demonstrates the efficacy of a combination therapy strategy involving the use of RNA interference to target the silencing of STAT3 and NANOG expression, in combination with the activity of xanthone-like compounds (MAG and GA) and their synthetic derivatives. This combination therapy strategy has the potential to yield promising results in terms of inhibiting cancer cell progression. The primary limitation of the preceding studies pertains to the utilisation of a single-cell line representing cervical cancer—HeLa cells. Nevertheless, the achievement of a satisfactory anti-proliferative, cytotoxic, and pro-apoptotic effect in this particular cell line does pave the way for further research prospects. In a broader context, it is proposed that the strategy be verified using a more diverse model of different cancer cell lines, as well as with different carriers for newly developed compounds with anticancer potential.

## 5. Conclusions

In summary, our study demonstrates that an RNAi-based knockdown of NANOG or STAT3 can make HeLa cells more sensitive to xanthone and synthetic xanthone derivative treatments, a potential avenue for further research. Moreover, in our study, we observed a decrease in NANOG expression in response to the presence of xanthones, which could be associated with their pro-oxidant activity.

## Data Availability

The raw data supporting the conclusions of this article will be made available by the authors on reasonable request.

## Abbreviations

The following abbreviations are used in this manuscript.

CSC	Cancer stem cells
DAPI	4′,6-diamidino-2-phenylindole dihydrochloride
DCF	2′,7′-dichlorofluorescein diacetate
DMSO	Dimethyl sulfoxide
D-PBS	Dulbecco’s Phosphate Buffered Saline
EdU	5-ethynyl-2′-deoxyuridine
EF	Enrichment factor
FBS	Foetal bovine serum
GA	Gambogic acid
GFP	Green fluorescent protein
H_2_DCFDA	2′,7′-dichlorodihydrofluorescein diacetate
HPV	Human Papillomavirus
LDH	Lactate dehydrogenase
MAG	α-mangostin
NAC	N-acetyl-L-cysteine
RNAi	RNA interference
ROR	HOX transcript antisense intergenic RNA
ROS	Reactive oxygen species
SCR	Scrambled non-targeting sequence
shRNA	Short hairpin RNA
siRNA	Small interfering RNA
STATs	Signal transducers and activators of transcription
